# Comprehensive Testing of Chemotherapy and Immune Checkpoint Blockade in Preclinical Cancer Models Identifies Additive Combinations

**DOI:** 10.3389/fimmu.2022.872295

**Published:** 2022-05-11

**Authors:** Nicola Principe, Wayne J. Aston, Danika E. Hope, Caitlin M. Tilsed, Scott A. Fisher, Louis Boon, Ian M. Dick, Wee Loong Chin, Alison M. McDonnell, Anna K. Nowak, Richard A. Lake, Jonathan Chee, Willem Joost Lesterhuis

**Affiliations:** ^1^National Centre for Asbestos Related Diseases, University of Western Australia, Perth, WA, Australia; ^2^School of Biomedical Sciences, University of Western Australia, Crawley, WA, Australia; ^3^Institute for Respiratory Health, Perth, WA, Australia; ^4^JJP Biologics, Warsaw, Poland; ^5^Telethon Kids Institute, Perth, WA, Australia; ^6^Medical School, University of Western Australia, Crawley, WA, Australia

**Keywords:** chemo-immunotherapy combinations, immune checkpoint blockade (ICB) therapy, T cells, proinflammatory cytokine, TNFa = tumor necrosis factor–a, IL-1b, fluorouracil (5-FU), cisplatin

## Abstract

Antibodies that target immune checkpoints such as cytotoxic T lymphocyte antigen 4 (CTLA‐4) and the programmed cell death protein 1/ligand 1 (PD-1/PD-L1) are now a treatment option for multiple cancer types. However, as a monotherapy, objective responses only occur in a minority of patients. Chemotherapy is widely used in combination with immune checkpoint blockade (ICB). Although a variety of isolated immunostimulatory effects have been reported for several classes of chemotherapeutics, it is unclear which chemotherapeutics provide the most benefit when combined with ICB. We investigated 10 chemotherapies from the main canonical classes dosed at the clinically relevant maximum tolerated dose in combination with anti‐CTLA-4/anti-PD-L1 ICB. We screened these chemo-immunotherapy combinations in two murine mesothelioma models from two different genetic backgrounds, and identified chemotherapies that produced additive, neutral or antagonistic effects when combined with ICB. Using flow cytometry and bulk RNAseq, we characterized the tumor immune milieu in additive chemo-immunotherapy combinations. 5-fluorouracil (5-FU) or cisplatin were additive when combined with ICB while vinorelbine and etoposide provided no additional benefit when combined with ICB. The combination of 5-FU with ICB augmented an inflammatory tumor microenvironment with markedly increased CD8^+^ T cell activation and upregulation of IFNγ, TNFα and IL-1β signaling. The effective anti‐tumor immune response of 5-FU chemo-immunotherapy was dependent on CD8^+^ T cells but was unaffected when TNFα or IL-1β cytokine signaling pathways were blocked. Our study identified additive and non-additive chemotherapy/ICB combinations and suggests a possible role for increased inflammation in the tumor microenvironment as a basis for effective combination therapy.

## Introduction

Drugs that block immune checkpoint receptors such as CTLA-4, PD-1 or PD-L1 have revolutionised cancer treatment, with durable anti-tumor responses observed in a subset of cancer patients (1, 2). However, the majority of patients treated with immune checkpoint blockade (ICB) demonstrate little or no benefit. Conventional chemotherapy remains standard treatment for many cancers. In addition to cytotoxic effects on cancer cells, many chemotherapeutics are immunostimulatory, capable of; inducing immunogenic cell death (3), increasing antigen cross-presentation (4), increasing immune cell infiltration (5), depleting immunosuppressive cells (6, 7), and altering expression of immune checkpoint ligands (8, 9). As these characteristics have been linked to ICB efficacy, some chemotherapeutics could potentially enhance anti-tumor immune responses when combined with ICB and therefore combination therapy warrants further investigation. Combination ICB and chemotherapy has shown efficacy in several cancer types. In fact, of the many different drug classes that have been combined with ICB, classical cancer chemotherapy remains one of the most successful (10). Particularly in thoracic cancers, chemotherapy/ICB combinations have shown efficacy, with FDA approval in non-small cell lung cancer (11) and small cell lung cancer (12), and with promising results in malignant pleural mesothelioma (13).

Although the effects of individual chemotherapeutics on discrete components of the immune system have been extensively described, a systematic analysis of how different chemotherapies combine with ICB *in vivo* is lacking, and the molecular mechanisms underlying additive chemo-immunotherapy combinations remains unknown. In this study, we systematically interrogated the therapeutic interaction between ICB and different canonical classes of cancer chemotherapeutics, given at maximum tolerated dose (MTD), in two preclinical cancer models, and mapped the molecular and cellular profiles of additive combinations, with the aim of prioritizing combinations to take forward into clinical trials.

## Materials and Methods

### Mice

Female BALB/c and C57BL/6 mice (RRID: IMSR_ARC:BC, RRID: IMSR_ARC:B6) were bred and maintained at the Animal Resource Centre (Murdoch, WA, Australia) or Harry Perkins Institute of Medical Research (Murdoch, WA, Australia). All mice used were between 8-10 weeks of age and were maintained under standard specific pathogen free housing conditions at the Harry Perkins Bioresources North Facility (Nedlands, WA, Australia). All experiments were conducted in accordance with the code of conduct of the National Health and Medical Research Council (NHMRC) of Australia, and under the approval of the Harry Perkins Institute of Medical Research Animal Ethics Committee (protocols AE029, AE100, AE179).

### Cell Lines

Murine mesothelioma cell lines AB1 (CBA, Cat# CBA-0144, RRID: CVCL_4403), AB1-HA (CBA, Cat# CBA-1374, RRID: CVCL_G361) and AE17 (CBA, Cat#CBA-0156, RRID: CVCL_4408) were derived as previously described (14, 15). Cell lines were maintained in RPMI 1640 (ThermoFisher Scientific, Scoresby VIC, Australia) supplemented with 20 mM HEPES, 0.05 mM 2-mercaptoethanol, 100 units/mL penicillin (CSL, Melbourne VIC, Australia), 50 μg/mL gentamicin (David Bull Labs, Kewdale VIC, Australia), 10% Newborn Calf Serum (NCS; ThermoFisher Scientific, Scoresby VIC, Australia) and 50 mg/mL of geneticin for AB1-HA only (G418; Life Technologies). Cells were cultured for a minimum of 4 passages after thawing before inoculation into mice. Cell lines were validated yearly by flow cytometry for MHC-I molecules H2‐K^b^ (consistent with C57BL/6) and H2‐K^d^ (consistent with BALB/c), and for fibroblast markers E-cadherin, epithelial cell adhesion molecule, and platelet-derived growth factor receptor α (negative). All cell lines were tested for Mycoplasma spp., every 3 months by PCR and found to be negative.

### Tumor Cell Inoculation

Cells were harvested when they reached 80% confluence. The right-hand flanks of mice were inoculated subcutaneously with 5 x 10^5^ tumor cells suspended in 100 µL of PBS. Mice were randomized prior to treatment, when tumors were palpable. Tumor dimensions (length and width) were measured with digital calipers by an investigator blinded for treatment allocation and tumor growth was represented as area (mm^2^).

### Chemotherapy, ICB and Antibody Treatments

Chemotherapy and ICB were administered on the same day, initiating treatment when tumors were approximately 20-25 mm^2^ in size. Chemotherapies were provided by Sir Charles Gardiner Pharmacy (Nedlands, WA, Australia) and was administered at the predetermined MTD as previously reported (16), except 5-FU which was administered at 75 mg/kg because MTD 5-FU with ICB caused severe toxicity (**Table S1**). Anti-CTLA-4 (clone 9H10, JJP Biologics) was dosed once at 100 μg/mouse and anti‐PD‐L1 (clone MIH5, JJP Biologics) was dosed 3 times with 2-day intervals at 100 μg/mouse (17). For depletion experiments, anti-CD4 (clone GK1.5, BioXcell), anti-CD8 (clone YTS 169, BioXcell) or anti-IL1β (clone B122, BioXcell) antibodies were administered 3 times with 3-day intervals at 100 μg/mouse with the first dose commencing 3 days before chemo‐immunotherapy. Anti-TNFα (clone XT3.11, BioXcell) was administered using the above schedule but at 2 mg/mouse. All treatments were diluted in sterile 0.9 % sodium chloride and administered intraperitoneally (i.p.) or intravenously (i.v.) as described in **Table S1**.

### Preparation of Single Cell Suspensions

Spleen and draining lymph nodes (DLNs) were digested with 1 mg/mL type IV collagenase (Worthington Biochemical) and 1 μg/mL DNase (Sigma Aldrich) in RPMI-1640 supplemented with 2% NCS and 20 mM HEPES for 25 minutes at room temperature. Red blood cells were lysed with Pharm Lyse (BD Biosciences). All cell suspensions were resuspended in EDTA‐BSS-NCS. Absolute numbers of leucocytes in DLNs were obtained using the Z2 Coulter Counter Analyzer (Beckman Coulter).

Tumors were processed using the Miltenyi Biotec mouse tumor dissociation kit, as per manufacturer’s protocol. Briefly, tumors were cut into 2-4 mm pieces and added to GentleMACs C tubes with 2.35 mL RPMI media supplemented with 10% NCS. Prioprietary enzyme mix was added, and samples mechanically digested using the GentleMACS Octo Dissociator 37C_M_TDK_2 protocol.

### Flow Cytometry

Three flow cytometry panels outlined in **Table S2** were used to characterize lymphoid and myeloid cell subsets. CD16/32 Fc block (eBioscience) and Zombie UV™ (Biolegend) viability dye were diluted in PBS and added to samples prior to surface antigen staining. All antibodies for surface staining were diluted in PBS + 2% NCS. Cells were permeabilized using the Foxp3/Transcription Factor Staining Buffer Set (eBioscience). Cells were washed with Permeabilization Buffer (eBioscience) and subjected to intracellular staining. Single stain and fluorescence minus-one (FMO) controls were also performed. To measure granzyme B (GzmB) and IFNγ, samples were incubated in Brefeldin A (Biolegend) for 4 hours at 37°C before antibody staining. Data was acquired using a BD LSRFortessa™ SORP with 50,000 live events collected per sample where possible. All flow cytometry analyses were completed using FlowJo™ Software version 10 (BD Biosciences). Summary of antibody concentrations and gating strategies are outlined (**Table S2**; **Figure S1**).

### Flow Cytometry Data Analysis

FCS files were subjected to automatic quality control of signal acquisition and dynamic range by the flowAI (v1.8) package using default parameters. Bad events (defined by negative outliers) were excluded, and manual gating was performed as outlined in **Figure S1**. For clustering analysis on the lymphoid cells, each sample was downsampled to 5,000 CD45^+^ cells using the DownSample (v3.1.0) package. All samples from all groups were then concatenated. The UMAP (v2.2) and Phenograph (v1.3) packages using default parameters (k = 30) were performed in FlowJo using the concatenated FCS file. Clusters were manually grouped into the final subsets described in **Figure 3**. Clusters were combined based on similar location on the UMAP plot and similar expression of key markers. Clusters that were CD45^+^ but had no expression of other phenotypic markers in the panel were colored grey and excluded from the analysis.

### Tumor Preparation for Bulk RNAseq

Whole tumors were harvested and stored in RNAlater (Life Technologies) at -80°C. RNA was extracted from frozen tumors using the RNeasy Plus Mini Kit and Tissue Ruptor (QIAGEN). RNA quality was confirmed on the Bioanalyzer (Agilent Technologies). Library preparation and sequencing (100-base pair single-end on an Illumina HiSeq platform) were performed by the Australian Genome Research Facility (Melbourne, VIC, Australia).

### Bulk RNAseq Analysis

Raw FASTQ files were aligned to the GRCm38/mm10 reference genome using Kallisto (18). Transcripts with low counts were removed and two count matrices were compiled using Tximport (19). The DESeq2 package (20) was used to identify differentially expressed genes (DEGs) between the following comparisons: PBS vs ICB, 5-FU, cisplatin, 5-FU+ICB or cisplatin+ICB; ICB alone vs 5FU+ICB or cisplatin+ICB, 5-FU alone vs 5-FU+ICB and cisplatin alone vs cisplatin+ICB. P values were adjusted for multiple comparisons using the Benjamini-Hochberg (B-H) method. A p value < 0.05 and a Log2 fold change cut-off of 0.5 were used to select DEGs. A full list of DEGs between each comparison can be found in **Supplementary File 1**.

Pathway analysis on up-and down-regulated DEGs between each comparison were performed using Enrichr (21). Over-representation of pathways from KEGG Mouse 2019 and Reactome 2016 databases were mapped using DEGs as input. The enrichment of upregulated ligands from the LINCS L1000 connectivity map were also analyzed using DEGs in Enrichr. Upstream regulator analysis was performed with DEGs and associated log fold changes as input, using the Ingenuity Systems program (22). Default settings were used and activation *Z*‐scores were used to determine the activation state of each upstream regulator. Those with activation *Z*-scores of ≥ 2 were considered ‘activated’ while activation *Z*-scores of ≤ -2 were considered ‘inhibited’. Upstream regulators included cytokines, transcription regulators, complexes, enzymes and kinases. For these analyses, p values were adjusted for multiple comparisons using the Benjamini-Hochberg method and p < 0.05 was considered significant.

Count data was scaled up to library size using Tximport (19) resulting in scaled transcripts per million (TPM) normalized count matrices. Heatmaps with unsupervised hierarchical clustering of the top 200 variable DEGs, determined by standard deviation were performed using the pheatmap package in R (v3.6). Gene set enrichment analysis (GSEA) was completed on the normalized gene expression data using 50 MSigDB hallmark gene sets on the Broad Institute software (23). Gene sets enriched with a FDR > 0.25 were considered significant. A total of 1000 permutations were performed, and all other default parameters were used. CIBERSORTx was used to identify immune cell populations in normalised RNAseq data as previously described (17).

### Statistical Analysis

Data are presented as mean ± SD. For flow cytometry experiments, statistical analyses were performed using Mann-Whitney U tests with multiple comparisons to compare between monotherapies and combination chemo/immunotherapy-treated samples using GraphPad Prism v8. Survival data were analyzed using Log-rank (Mantel-Cox) test in GraphPad Prism v8. To compare combination treatments to monotherapy controls, hazard ratios (HR) were calculated using logrank analysis of survival curves to determine agonistic or antagonistic effects. To further define additive interactions, as described before (24), HR was calculated for each treatment group compared to PBS or best monotherapy treated controls. Additive effects were defined as HR(combination) < [HR(combination) - HR(mono 1) - HR(mono 2) + 1]. Results of these analyses are displayed in **Table S3**.

## Results

### 5-FU and Cisplatin Generate Robust Anti-Tumor Responses When Combined With ICB

**Figure 1:**
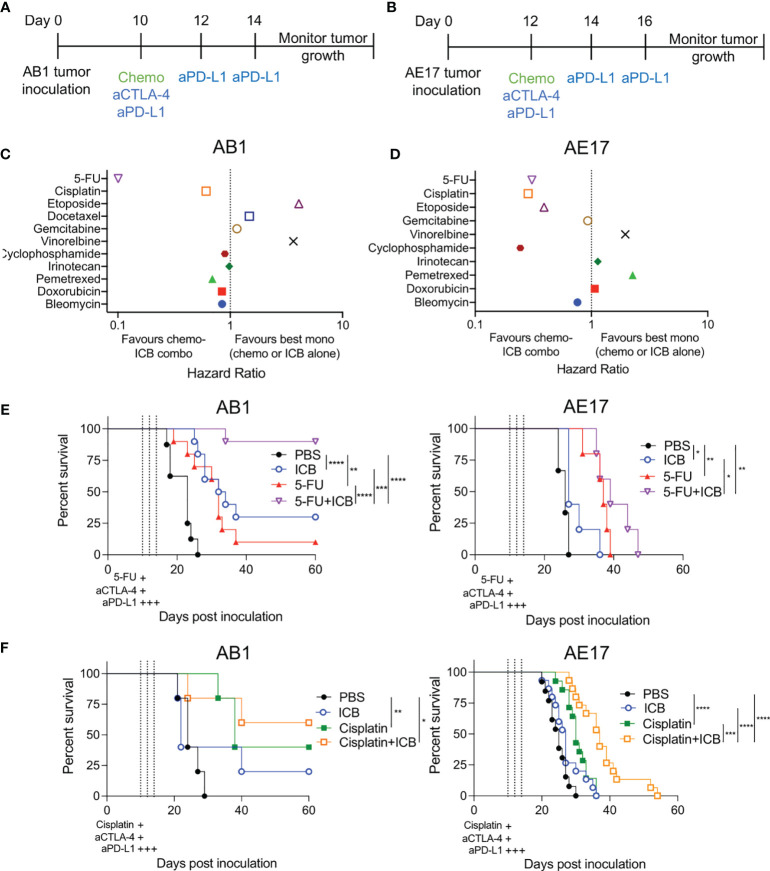
Different combinations of chemotherapy and immune checkpoint blockade (ICB) demonstrate additive and antagonistic responses. **(A, B)** Treatment schedule for mice inoculated with AB1 **(A)** or AE17 **(B)** mesothelioma cell lines. **(C, D)** Hazard ratio (HR) analysis of survival plots comparing combination chemotherapy and ICB (anti-CTLA‐4/anti‐PD-L1) to the best performing monotherapy in AB1 **(C)** and AE17 **(D)**. HR is defined as the risk of a negative (death) outcome occurring in one group at the next instance of time, compared to another group at the same time. A lower ratio i.e., less than 1 indicates a higher rate of survival in the chemo-immunotherapy combination compared to monotherapy. **(E)** Survival curves of 5-FU chemo-immunotherapy combinations in AB1 (left; n = 8-10 per group, two pooled experiments) and AE17 (right; n = 5 per group, one experiment). **(F)** Survival curves of cisplatin chemo-immunotherapy combinations in AB1 (left; n = 5 per group, one experiment) and AE17 (right; n = 13-15 per group, two pooled experiments). Mantel-Cox survival test; *P < 0.05, **P < 0.01, ***P < 0.001, ****P < 0.0001.

The addition of ICB with chemotherapy regimens are increasingly being trialed in the clinic to improve patient outcomes (25). However, the impact of individual chemotherapies on ICB efficacy remains unclear. To assess anti-tumor responses of chemotherapy when combined with ICB *in vivo*, we screened 10 chemotherapeutics from different canonical classes in combination with anti‐CTLA‐4/anti‐PD-L1 antibodies in two murine mesothelioma models (**Figures 1A, B**). As there is a difference in the therapeutic response to the different chemotherapies (**Figures S2, 3**), we compared survival of the combination therapy with survival of the best monotherapy (either chemotherapy or ICB alone) and plotted each as a hazard ratio (HR). ICB alone induced complete tumor regression in 0-30% of AB1 tumor bearing animals but not in the AE17 model. When combined with ICB, all tested chemotherapies had varying effects on anti-tumor efficacy across the two models (**Figures 1C, D**, **S2, 3**, **Table S3**). Gemcitabine, irinotecan, doxorubicin and bleomycin provided no benefit when combined with ICB in either AB1 (HR = 1.14, 0.979, 0.845, 0.845, 0.692 respectively) or AE17 (HR = 0.929, 1.14, 1.07, 0.759 respectively). ICB provided no further benefit when added to cyclophosphamide in AB1 (HR = 0.895), but the combination significantly improved median survival in AE17 (HR = 0.244). The combination of vinorelbine or etoposide with ICB was antagonistic in AB1 (HR = 3.65, 4.08) but had no effect in AE17 (HR = 1.96, 0.391). The reverse was the case for pemetrexed (HR = 0.692 in AB1, 2.261 in AE17).

The combination of 5-FU with ICB (5-FU+ICB) resulted in robust anti-tumor responses in the AB1 model (HR = 0.101), with significant increase in median survival compared to both 5-FU (p = 0.0001) and ICB (p = 0.005) monotherapy. Complete tumor regression occurred in >80% of 5-FU+ICB treated animals, compared to 0-20% or 20-30% complete responders in 5-FU or ICB monotherapy, respectively (**Figure 1E**). 5‐FU+ICB was also additive in AE17 (HR = 0.308), with an increase in median survival compared to the monotherapies (5-FU: p = 0.199, ICB: p = 0.0142). Cisplatin and ICB (cisplatin+ICB) were additive in both AB1 (HR = 0.610) and AE17 (HR = 0.286) (**Figure 1F**). In AE17, cisplatin+ICB significantly increased median survival compared to cisplatin (p = 0.0025) and ICB (p<0.0001) monotherapy. Taken together, these data demonstrate that different chemotherapies display additive, antagonistic or neutral interactions with ICB and that these interactions could be variable between models.

### Combination ICB With 5-FU or Cisplatin Induces Profound Expansion of Tumor Draining Lymph Nodes

**Figure 2:**
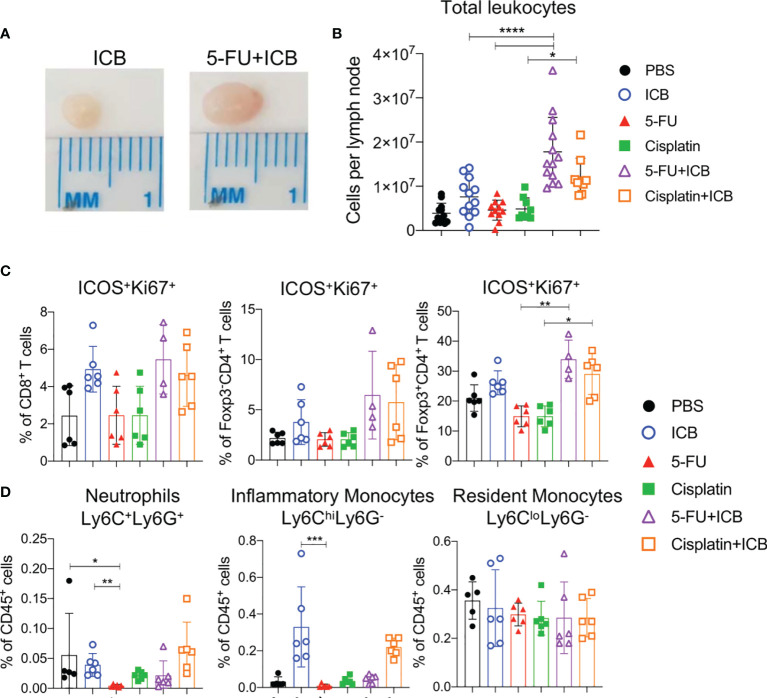
5-FU and cisplatin chemotherapy in combination with ICB causes expansion of T cells in tumor draining lymph nodes. **(A)** Representative images of tumor draining lymph nodes from AB1 tumor bearing mice after ICB (left) or 5-FU+ICB (right). **(B)** Absolute numbers of leukocytes, **(C)** proportions of activated (ICOS^+^Ki67^+^) CD8^+^, CD4^+^Foxp3^-^ (helper) and CD4^+^Foxp3^+^ (regulatory; T_regs_) T cells, and **(D)** proportions of neutrophils (CD11b^+^Ly6C^+^Ly6G^+^), inflammatory monocytes (CD11b^+^Ly6C^hi^Ly6G^-^), resident monocytes (CD11b^+^Ly6C^lo^Ly6G^-^) in DLNs of different treatment groups. Data represented as mean ± SD, summary of three independent experiments. Mann-Whitney U test corrected for multiple comparisons; *P < 0.05, **P ≤ 0.01, ***P ≤ 0.001, ****P ≤ 0.0001.

To understand how 5-FU and cisplatin enhance the anti-tumor immune response when combined with ICB, we first analyzed tumor draining lymph nodes (DLNs) and spleens from treated, tumor-bearing mice (**Figure S4A**). We focused on the AB1 model because 5-FU and cisplatin produced the most robust responses in this model. DLNs from both cisplatin+ICB and 5-FU+ICB groups were larger in size relative to monotherapy groups (**Figures 2A, B**). The absolute number of leucocytes in DLNs of 5‐FU+ICB treated animals was significantly greater compared to DLNs from either 5-FU (p < 0.0001) or ICB (p < 0.0001) monotherapies. Cisplatin+ICB treated DLNs contained a significantly greater number of leucocytes compared to those treated with cisplatin alone (p = 0.015). The proportions of CD8^+^, CD4^+^Foxp3^-^ and CD4^+^Foxp3^+^ T cells in DLNs and spleens were similar between all treatment groups (**Figures S4B, C**). We observed increased proportions of activated, proliferating CD4^+^Foxp3^+^ICOS^+^Ki67^+^ T cells in DLNs (**Figure 2C**) and spleens (**Figure S4D**) of combination treated animals compared to 5-FU or cisplatin chemotherapy alone. We found minor depletion of neutrophils (CD11b^+^Ly6C^+^Ly6G^+^) and inflammatory monocytes (CD11b^+^Ly6C^hi^Ly6G^-^) in DLNs (**Figure 2D**) and spleens (**Figures S5A, B**) in 5-FU treated groups as reported previously (26). These data suggest that additive chemo-immunotherapy combinations induce a profound expansion of leucocytes in tumor DLNs.

### Additive Chemo-Immunotherapy Combinations Increase the Frequency of Intratumoral T Cells

**Figure 3:**
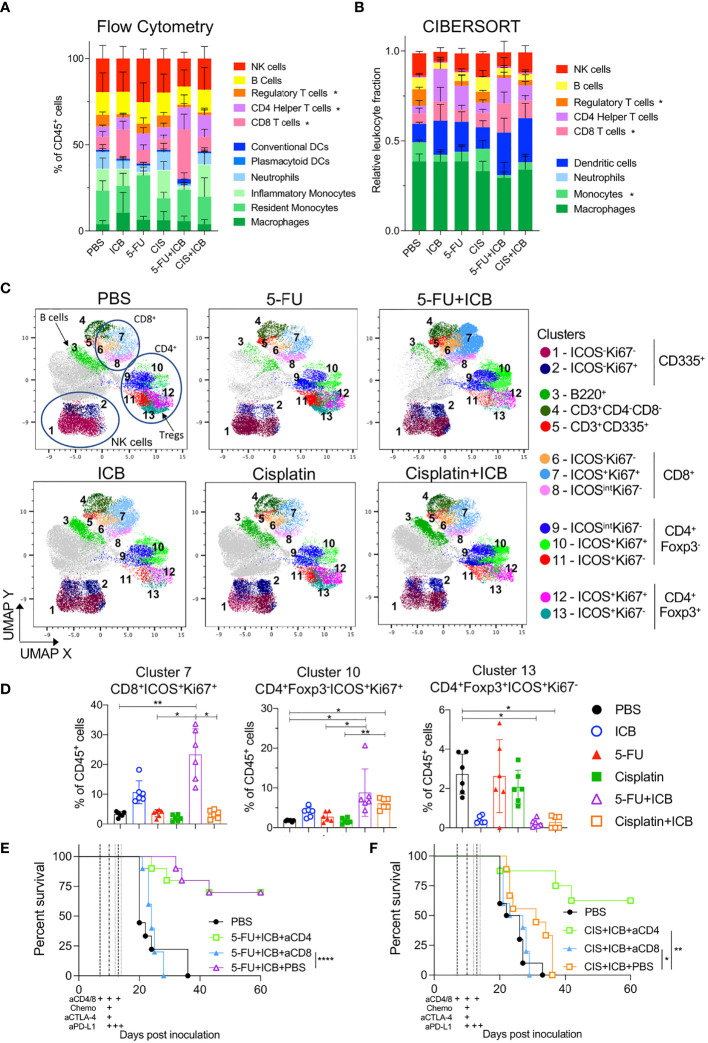
Tumors of additive chemo-immunotherapy combinations are enriched for activated CD8^+^ and CD4^+^ T cells. **(A, B)** Summary of lymphoid and myeloid immune cell proportions in chemo-immunotherapy treated tumors analyzed using flow cytometry **(A)** and CIBERSORTx **(B)** from bulk RNAseq. * indicates p < 0.05 for that cell type between chemo-immunotherapy and PBS controls. **(C)** UMAP plots of clustered CD45^+^ cells from flow cytometry data for each treatment group. Cells are colored by Phenograph clusters and annotated by expression of phenotypic markers in legend. Cells colored in grey had no expression of other phenotypic markers in panel and were excluded from analysis. **(D)** Frequencies of cells from clusters 7.10,13 in all chemotherapy and/or ICB treated tumors. **(E, F)** Survival curves of AB1-HA tumor bearing mice treated with 5-FU+ICB **(E)** and cisplatin+ICB **(F)** with or without anti-CD4 or anti-CD8 depletion antibodies. Dotted lines indicate when therapies were administered. Data shown as mean ± SD, flow cytometry data is summary of two independent experiments (n = 6 per group), RNAseq data (n = 5 per group except PBS and cisplatin; n = 4 per group), *in vivo* tumor growth data is summary of two independent experiments (n = 10 per group). Mann-Whitney U test corrected for multiple comparisons and Mantel-Cox survival test; *P < 0.05, **P ≤ 0.01, ****P ≤ 0.0001.

To determine if there were specific intratumoral immune cells involved in additive chemo‐immunotherapy combinations, we first characterized tumor infiltrating immune cell populations of 5-FU and cisplatin chemo/immunotherapy-treated animals by flow cytometry and CIBERSORT analysis of bulk RNAseq data. In terms of overall immune cell populations, we did not see consistent significant differences between combination therapy to chemotherapy or ICB alone (**Figures 3A, B**). Tumors from 5-FU+ICB treated mice displayed increased CD8^+^ T cell infiltration as identified by both CIBERSORT and flow cytometry analyses (p = 0.04; **Figures 3A, B**, **S6A, B**). The number of intratumoral CD4^+^ helper T cells were significantly greater in cisplatin+ICB treated mice compared to cisplatin only (p = 0.01; **Figures 3A**, **S6A**). The frequency of CD4^+^Foxp3^+^ regulatory T cells (T_regs_) was reduced in tumors from 5-FU+ICB treated mice compared to PBS controls in both data sets. T_regs_ in cisplatin+ICB treated tumors were significantly reduced compared to PBS controls in flow cytometry data (p = 0.02; **Figures S6A, B**). The frequency of monocytes significantly decreased in 5-FU+ICB tumors compared to PBS in the CIBERSORT analysis (p = 0.028) but did not reach statistical significance in the flow cytometry data (**Figures S6C, D**).

We further characterized the activation status of tumor infiltrating lymphocytes (TILs). Dimensional reduction analyses on CD45^+^ cells using UMAP and Phenograph produced 13 distinct phenotypic clusters (**Figure 3C**). The frequency of activated (ICOS^+^) and proliferating (Ki67^+^) CD8^+^ T cells (cluster 7) was significantly greater in tumors from 5‐FU+ICB treated mice (23.3 ± 8.69%) compared to 5-FU alone (3.62 ± 1.21%; p = 0.03, **Figure 3D**). We also characterized GzmB expression and IFNγ secretion from CD8^+^ T cells and found a significant increase in the total number of IFNγ^+^ CD8^+^ TILs in chemo‐immunotherapy treated tumors compared to 5-FU treated tumors (**Figure S7**). Tumors from both cisplatin+ICB and 5-FU+ICB treated mice were enriched with increased CD4^+^Foxp3^-^ICOS^+^Ki67^+^ T cells (cluster 10) compared to chemotherapy alone (5‐FU+ICB vs 5-FU; 8.81 ± 5.94% vs 2.74 ± 1.22%; p = 0.04; cisplatin+ICB vs cisplatin; 6.09 ± 1.37% vs 1.69 ± 0.70%; p = 0.005). Activated tumor-infiltrating T_regs_ (CD4^+^Foxp3^+^ICOS^+^Ki67^-^; cluster 13) were also significantly reduced in both combination chemo‐immunotherapy treated groups (5-FU+ICB: 0.25 ± 0.20%; cisplatin+ICB: 0.29 ± 0.31%) compared to PBS controls (2.73 ± 1.01%; p = 0.015; p = 0.014 respectively) (**Figure 3D**).

As both 5-FU and cisplatin chemo-immunotherapies increased activated and proliferating CD8^+^ and CD4^+^Foxp3^-^ T cells, we depleted CD8^+^ or CD4^+^ T cells throughout the treatment schedule in AB1-HA tumor-bearing mice to test whether these cells were required for effective chemo‐immunotherapy. Depleting CD8^+^ T cells significantly abrogated the anti-tumor effect of both chemo-immunotherapy combinations (5-FU+ICB: p < 0.0001; cisplatin+ICB: p = 0.026) **(Figures 3E, F)**. In comparison, depleting CD4^+^ T cells had no significant impact on the efficacy of 5-FU+ICB combination therapy (P = 0.45), but significantly improved survival in the cisplatin+ICB combination group (p = 0.0014) **(Figures 3E, F)**. These data demonstrate that 5‐FU/cisplatin combined with ICB enhanced activation of intratumoral CD8^+^ and CD4^+^ T cells, and the anti‐tumor response for these chemo-immunotherapy combinations were dependent on CD8^+^ T cells.

### Inflammatory T Cell-Driven Pathways Are Enriched in Additive Chemo-Immunotherapy Combinations

**Figure 4:**
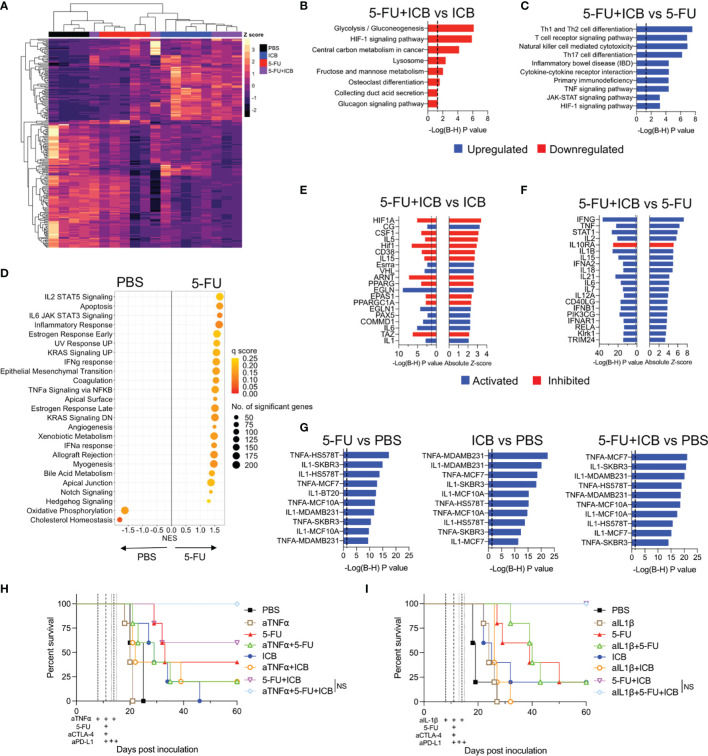
5-FU-based chemo-immunotherapy upregulates immune-associated pathways and downregulates hypoxia and glycolysis pathways. **(A)** Unsupervised hierarchical clustering of the top 200 differentially expressed genes from 5-FU+ICB treatment groups. **(B)** Downregulated KEGG pathways in 5-FU+ICB compared to ICB treated tumors. **(C)** Top 10 upregulated KEGG pathways in 5-FU+ICB when compared 5-FU. Multiple comparisons corrected using Bonferonni-Hochberg method. Significance denoted by P < 0.05. **(D)** GSEA displaying top hallmark gene sets significantly (q < 0.25) enriched in 5-FU compared to PBS. A positive normalized enrichment score (NES) indicates that specific gene set is enriched in a 5-FU treated tumor compared to PBS. **(E, F)** Graphs displaying the top 20 upstream regulators in 5-FU+ICB compared to ICB **(E)** or 5-FU **(F)** treated tumors. Upstream regulators are colored by the activation *Z*-score. Regulators with a *Z-*score ≥ 2 are activated and are displayed in blue. Regulators with a *Z*-score ≤ -2 are inhibited and are displayed in red. **(G)** Top 10 most significantly upregulated LINCS L1000 gene signatures in 5-FU, ICB and 5-FU+ICB in comparison to PBS treated tumors. Multiple comparisons corrected using Bonferonni‐Hochberg method. Significance denoted by P < 0.05. **(H–I)** Survival curves of AB1‐HA tumor bearing mice treated with anti-TNFα **(H)** or anti-IL-1β **(I)** blocking antibodies, 5-FU, ICB or 5-FU+ICB therapies. Data represents one experiment (n = 5 per group). Mantel‐Cox survival test. B-H, Bonferonni-Hochberg.

To further elucidate the molecular and cellular pathways inducing the robust anti-tumor responses in effective chemo-immunotherapies, we compared the gene expression profiles of additive chemo‐immunotherapy combinations and monotherapy-treated tumors. Unsupervised hierarchical clustering of the top 200 most variable DEGs for each chemo-immunotherapy compared to monotherapy controls (**Figures 4A**, **S8A**) demonstrated that ICB alone, or in combination with chemotherapy was driving most of the differences in gene expression. 5-FU alone was also clearly separated from PBS and ICB treated tumors (**Figure 4A**) whereas cisplatin alone was similar to PBS treated tumors (**Figure S8A**).

To determine the biological relevance of the DEGs identified in the additive chemo-immunotherapy combinations, we examined the over-representation of pathways using KEGG and Reactome databases. We first compared the combination therapies with ICB monotherapy. A total of 330 genes were differentially expressed between 5-FU+ICB and ICB, which were associated with a downregulation of pathways involved in glucose metabolism and hypoxia by 5-FU+ICB (**Figures 4B**; **S8B**). Only 55 genes were differentially expressed between cisplatin+ICB and ICB, and pathway analysis did not identify a common biological pathway associated with these genes (data not shown).

We next compared each combination therapy with their respective chemotherapy alone and identified 779 DEGs comparing cisplatin+ICB with cisplatin monotherapy, and 536 DEGs between 5-FU+ICB and 5-FU monotherapy. Pathway analysis of DEGs demonstrated that both chemo-immunotherapy combinations significantly upregulated immune-related pathways involving immune cell differentiation, signaling and cytotoxicity compared to either respective chemotherapy alone, suggesting enhancement of the immunological activity of ICB (**Figures 4C**; **S8C–E**). These results were confirmed using GSEA (23), applying the curated Hallmark gene sets to the gene expression profiles (**Figure S8F**). Interestingly, 5-FU treated tumors were enriched with multiple immune related gene sets compared to PBS controls (**Figure 4D**), similar to the ICB gene expression profile reported previously (17). Together these results indicate that 5-FU may be further enhancing the immunostimulatory effects of ICB, generating a robust anti-tumor immune response seen for the combination therapy.

Having characterized molecular pathways that were associated with additive chemo-immunotherapy combinations, we next sought to identify key targets that could modulate the anti-tumor immune response. We focused on the 5-FU chemo-immunotherapy combination as it produced the most robust anti-tumor immune response *in vivo* (**Figure 1**). We performed upstream regulator analysis to identify key transcriptional regulators of molecular pathways enriched in 5-FU chemo-immunotherapy. In comparison to ICB, 5-FU+ICB induced a gene expression signature indicative of inhibition of upstream regulators involved in HIF1 signaling (HIF1A, CSF1, ARNT), peroxisome signaling (PPARG, PPARGC1A) and activation of upstream regulators IL-1 and IL-6 (**Figure 4E**). IFNγ, TNFα, IL-2, STAT1 and IL-1β were the top activated upstream regulators in 5-FU+ICB compared to 5-FU monotherapy (**Figure 4F**). We also analyzed the data using the LINCS L1000 connectivity map which measured the expression of over 3000 genes in eight different cell lines following exposure to defined ligands. The gene expression profiles of 5-FU, ICB and 5-FU+ICB treated tumors, were enriched for IL-1 and TNFα-induced genes (**Figure 4G**).

To test if TNFα or IL-1β cytokine signaling pathways were required to produce the robust anti-tumor immune response found when 5-FU is added to ICB, we administered TNFα or IL‐1β blocking antibodies throughout the 5-FU+ICB treatment schedule in AB1-HA tumor bearing mice (**Figures 4H, I**). The efficacy of the 5-FU+ICB combination was unaffected when either pro-inflammatory cytokine was depleted. There was also no significant difference in survival for the 5-FU or ICB monotherapies when TNFα or IL-1β were blocked. (**Figure 4H**) (5-FU+ICB vs 5-FU+ICB+aTNFa, P = 0.136; 5‐FU+ICB vs 5-FU+ICB+aIL-1β, P > 0.999). This indicates that whilst TNFα and IL-1 signaling were significantly enriched in 5-FU chemo-immunotherapy treated tumors, the robust anti-tumor response produced by this additive chemo-immunotherapy is likely to be dependent on the combination of multiple molecular pathways.

## Discussion

In this study, we analyzed the *in vivo* anti-tumor effects of 10 different chemotherapies in combination with anti-CTLA-4 and anti-PD-L1 ICB to identify effective chemo‐immunotherapy combinations. We found that the addition of 5-FU or cisplatin to ICB significantly improved survival compared to either monotherapy alone in two murine cancer models. Importantly, no chemo-immunotherapy combination decreased overall survival compared to ICB alone, suggesting no antagonistic effects of tested chemotherapies.

Immunogenic chemotherapies such as vinorelbine, etoposide, cyclophosphamide and gemcitabine induced robust anti-tumor responses alone. While we only found 5-FU or cisplatin improved the efficacy of ICB, other studies have identified that vinorelbine and etoposide synergized with anti‐CTLA-4 (27) and anti-PD-L1 (28) respectively. In addition, cyclophosphamide and gemcitabine have been previously shown to enhance CD8^+^ T cell infiltration in tumors and deplete immunosuppressive cells (29, 30), but preclinical studies combining these chemotherapies with ICB have provided conflicting results (27–29, 31). These discrepancies may not only be due to different cancer models but also chemotherapy dosing and scheduling. For example, we previously established that multiple lower doses of gemcitabine (240 mg/kg) were synergistic with ICB in the AB1 tumor model (32), whereas MTD gemcitabine (700 mg/kg) did not provide additional benefit in this study. 5‐FU at the previously reported MTD of 125 mg/kg (16) could not be administered with ICB without severe toxicity so a lower dose (75 mg/kg) was used which may have impacted the additive effect found with this combination. In addition, we investigated chemo-immunotherapy combinations in subcutaneous models of mesothelioma which may not fully recapitulate the tumor microenvironment in the pleural mesothelium. However, orthotopic models of mesothelioma are technically challenging and response rates to ICB and chemotherapy monotherapy in our subcutaneous models are similar to responses found in mesothelioma patients (33, 34).

We also administered ICB and MTD chemotherapy concurrently. Although staggering the ICB and chemotherapy doses have been explored previously (29, 35–37), it is difficult to separate the immunogenic effects of therapy from tumor size, particularly when one treatment has substantially reduced the tumor size before the addition of the next therapy. Administering chemotherapy before ICB could induce a highly inflammatory tumor microenvironment, sensitize tumor cells to cytotoxic T cell killing (38, 39), priming a tumor to be more responsive to ICB. Dosing and scheduling are potential factors that could affect the chemotherapy-induced immune response, and remain an important area of research, going forward.

Our study focused on the additive mechanisms of MTD 5-FU and cisplatin to ICB as they produced the most robust anti-tumor responses in our models. 5-FU has shown to be additive when combined with anti-PD-1/PD-L1 ICB in other pre-clinical models (35, 36, 40), whereas cisplatin chemotherapy synergized with anti‐PD‐1/PD‐L1 or anti‐CTLA-4 in some models, but not others (41, 42). Platinum‐based chemotherapy has been shown to be a very effective combination with ICB in patients (10). In addition, multiple ongoing clinical trials are analyzing the efficacy of combination multi-modal chemotherapy, (including 5-FU and cisplatin) with ICB, particularly for patients with colorectal and bladder cancer (NCT03202758, NCT02658214, NCT04241185, NCT03775265, NCT02912559). Increased numbers of activated CD8^+^ T cells at the tumor site together with depletion of immunosuppressive cells (T_regs_ and MDSCs) were key immunological effects of 5-FU and cisplatin chemo-immunotherapy combinations in our study and others (40, 42).

Gene signatures associated with hypoxia and metabolism, in particular HIF-1α and glycolysis pathways were downregulated in tumors from mice treated with the 5-FU+ICB combination compared to monotherapy. HIF-1α signaling regulates chemotherapy-resistance, and the differentiation of immunosuppressive myeloid-derived suppressor cells (MDSCs) (43). Both AB1 and AE17 tumors display hypoxic regions *in vivo* (44), and chemo-immunotherapy could have altered tumor hypoxia. It is also possible that combination 5-FU and ICB alter tumor immunosuppressive cells through HIF-1α mediated pathways, resulting in decreased MDSCs and T_regs_ observed in our study. Others have found that inhibition of the HIF-1α pathway improves the anti-tumor effect of 5-FU (45), and improves ICB responses (46) in preclinical models. There are numerous small molecule drugs that inhibit different parts of the HIF-1α signaling pathway, but the clinical efficacy of HIF-1α inhibitory drugs in cancer have been limited thus far (47).

Recent studies have also highlighted the importance of glucose metabolism in T cell activation and proliferation in response to a T cell receptor mediated stimulus. PD-1 and CTLA-4 signaling inhibit glycolysis in CD4^+^ and CD8^+^ T cells *in vitro*, preventing rapid proliferation and differentiation into effector cells (48). It is therefore counterintuitive that glycolysis pathways would be downregulated in the most efficacious chemo-immunotherapy combination from our study. However, a caveat of our study is that RNAseq of bulk tumors did not allow us to separate the metabolic effects of chemo-immunotherapy on tumor versus immune cells. The metabolic competition between tumor cells and T cells has been well described (49), and reduced glycolysis within tumors have been observed, particularly with PD-L1 blockade.

TNFα and IL-1β signaling associated genes were upregulated in 5-FU+ICB treated tumors. However, antibody neutralization experiments showed that these signaling pathways were not necessary for complete tumor regression. In fact, neutralization of TNFα further improved the efficacy of 5-FU+ICB. This is in line with multiple reports demonstrating that disruption of TNFα or IL-1R/IL-1β signaling by either blocking antibodies or deficient mouse models improves the anti-tumor immune response in combination with 5-FU (50, 51) or ICB (52, 53). These results highlight the complexity, and redundancy of different pro‐inflammatory cytokines in mediating anti‐tumor immunity. As TNFα and IL-1β inhibitors are now available to treat severe ICB induced immune related adverse events (54), it is encouraging that blocking these pathways did not diminish the anti-tumor responses of chemo-immunotherapy in preclinical models.

Our study provides a resource and starting point for future studies to interrogate the mechanisms of combination ICB and chemotherapy. 5-FU and cisplatin treated tumors had vastly different gene expression profiles, suggesting additive mechanisms could be different for other chemotherapies. 5‐FU is currently not used clinically for mesothelioma, and the results with this chemotherapy may therefore be particularly applicable to other cancers. However, understanding additive mechanisms is important to develop novel strategies to phenocopy a responding tumor microenvironment, and improve anti‐tumor responses of chemotherapy and/or immunotherapy.

## Data Availability Statement

The original contributions presented in the study are publicly available. This data can be found here: https://www.ncbi.nlm.nih.gov/geo/query/acc.cgi?acc=GSE188481.

## Ethics Statement

The animal study was reviewed and approved by Harry Perkins Institute of Medical Research Animal Ethics Committee.

## Author Contributions

NP and WA performed all mouse experiments and wrote manuscript. NP, WA, CT, and DH performed mouse experiments. NP and WC analysed RNA sequencing data. NP and AM analysed flow cytometry data. ID assisted with statistical analysis. LB provided critical reagents. RL, AN, AM, SF, JC, and WL interpreted experiments and critically revised the manuscript. JC and WL shared the design of the study, supervised the project and edited the manuscript. All authors contributed to the article and approved the submitted version.

## Funding

This work was funded by NHMRC grant 1067113. NP was supported by UWA Richard Walter Gibbon Medical Research and Cancer Council WA scholarships. JC was supported by grants and fellowship from the UWA Raine Foundation, Cancer Council WA, WA Department of Health, and iCare Dust Diseases Board. WL was supported by fellowships from the Simon Lee Foundation, NHMRC and Cancer Council WA. The National Centre for Asbestos Related Diseases receive funding through the NHMRC Centres of Research Excellence scheme.

## Conflict of Interest

LB was employed by JJP Biologics.

The remaining authors declare that the research was conducted in the absence of any commercial or financial relationships that could be construed as a potential conflict of interest.

## Publisher’s Note

All claims expressed in this article are solely those of the authors and do not necessarily represent those of their affiliated organizations, or those of the publisher, the editors and the reviewers. Any product that may be evaluated in this article, or claim that may be made by its manufacturer, is not guaranteed or endorsed by the publisher.
