# Supplementary material for: Comprehensive Testing of Chemotherapy and Immune Checkpoint Blockade in Preclinical Cancer Models Identifies Additive Combinations
Source: Front Immunol. 2022 May 11;13:872295. doi: 10.3389/fimmu.2022.872295 (PMC9132586; doi:10.3389/fimmu.2022.872295)
Supplement: Supplementary file 1 [file DataSheet_1.docx]

Supplementary Material

| **Chemotherapy** | **Dose** | **Administration route** |
| --- | --- | --- |
| 5-Fluorouracil (5-FU) | 75 mg/kg | i.v. |
| Cisplatin | 6 mg/kg | i.p. |
| Cyclophosphamide (Cyclo) | 300 mg/kg | i.p. |
| Docetaxel | 130 mg/kg | i.p. |
| Doxorubicin | 10 mg/kg | i.p. |
| Etoposide | 75 mg/kg | i.p. |
| Gemcitabine | 700 mg/kg | i.p. |
| Irinotecan | 240 mg/kg | i.p. |
| Pemetrexed | 600 mg/kg | i.p. |
| Vinorelbine | 10 mg/kg | i.p. |

**Table S1. Doses of chemotherapies**

*i.p. – intraperitoneal, i.v. - intravenous

**Table S2. Flow cytometry panels.**

| **Fluorochrome** | **Antigen** | **Dilution** | **Clone** | **Supplier** | **Catalog No.** | **RRID** | **Panel** |
| --- | --- | --- | --- | --- | --- | --- | --- |
| FITC | CD45 | 1:200 | REA737 | Miltenyi Biotec | 130-110-796 | AB_2658216 | 1 |
| BUV395 | CD3 | 1:500 | 145-2C11 | BD | 103040 | AB_2738278 | 1,3 |
| BV480 | CD8a | 1:250 | 53-6.7 | BD | 566096 | AB_2739500 | 1,3 |
| AF647 | Foxp3 | 1:100 | 150D | Biolegend | 320013 | AB_439749 | 1 |
| BV786 | CD4 | 1:200 | GK1.5 | BD | 563331 | AB_563331 | 1 |
| PE-Cy7 | ICOS | 1:200 | C398.4A | Biolegend | 313519 | AB_10641939 | 1 |
| PE | Ki67 | 1:100 | B56 | BD | 556027 | AB_2266296 | 1 |
| BV711 | CD335 | 1:200 | 29A1.4 | Biolegend | 137621 | AB_2563289 | 1 |
| APCVio770 | B220 | 1:100 | REA755 | Miltenyi Biotec | 130-110-849 | AB_2658286 | 1 |
| BUV395 | CD45 | 1:250 | 30-F11 | BD | 564279 | AB_2651134 | 2 |
| BV510 | Ly6C | 1:400 | HK1.4 | Biolegend | 128033 | AB_2562351 | 2 |
| eV605 | CD11c | 1:200 | N418 | Biolegend | 117333 | AB_11204262 | 2 |
| PE | Siglec-F | 1:400 | REA798 | Miltenyi Biotec | 130-112-332 | AB_2653439 | 2 |
| APC | CD11b | 1:400 | REA592 | Miltenyi Biotec | 130-109-364 | AB_2654646 | 2 |
| AF700 | Ly6G | 1:400 | 1A8 | Biolegend | 127621 | AB_10640452 | 2 |
| APC-ef780 | MHCII | 1:200 | M5/114.15.2 | BD | 47-5321-80 | AB_1548792 | 2 |
| Zombie UV | FVD | 1:4000 |  | Biolegend | 423107 |  | 1,2.3 |
| BUV737 | CD4 | 1:1000 | RM4-5 | BD | 612844 | AB_2870166 | 3 |
| Pacific Blue | GzmB | 1:200 | GB11 | Biolegend | 515407 | AB_2562195 | 3 |
| PE-Cy7 | IFNg | 1:250 | XMG1.2 | eBioscience | 25-7311-82 | AB_469680 | 3 |
| APC-Cy7 | CD45 | 1:1000 | 30-F11 | BD | 561037 | AB_10563075 | 3 |

*Panel 1 – lymphoid, panel 2 – myeloid, panel 3 - cytokine

**Table S3. Hazard ratios (HR) for chemo-immunotherapy combinations in AB1 and AE17.**

| **AB1** | | | | | | |
| --- | --- | --- | --- | --- | --- | --- |
| **Chemotherapy** | **Best mono** | **HR Chemo vs PBS** | **HR ICB vs PBS** | **HR Combo vs PBS** | **HR Combo vs best mono** | **Additive Index** |
| 5-FU | ICB | 0.243 | 0.216 | 0.0372 | 0.101 | 0.578 |
| Cisplatin | Cisplatin | 0.179 | 0.690 | 0.204 | 0.610 | 0.335 |
| Pemetrexed | Pemetrexed | 0.455 | 0.690 | 0.313 | 0.692 | 0.616 |
| Doxorubicin | Doxorubicin | 0.326 | 0.690 | 0.177 | 0.845 | 0.608 |
| Bleomycin | Bleomycin | 0.243 | 0.188 | 0.218 | 0.845 | 0.786 |
| Cyclo | Cyclo | 0.103 | 0.209 | 0.103 | 0.895 | 0.792 |
| Irinotecan | ICB | 0.684 | 0.209 | 0.187 | 0.979 | 0.294 |
| Gemcitabine | Gemcitabine | 0.179 | 0.222 | 0.288 | 1.15 | 0.886 |
| Docetaxel | ICB | 0.247 | 0.209 | 0.223 | 1.48 | 0.768 |
| Vinorelbine | Vinorelbine | 0.088 | 0.222 | 0.236 | 3.65 | 0.925 |
| Etoposide | Etoposide | 0.089 | 0.222 | 0.179 | 4.08 | 0.869 |
| **AE17** | | | | | | |
| **Chemotherapy** | **Best mono** | **HR Chemo vs PBS** | **HR ICB vs PBS** | **HR Combo vs PBS** | **HR Combo vs best mono** | **Additive Index** |
| Cyclo | Cyclo | 0.220 | 0.522 | 0.206 | 0.244 | 0.460 |
| Cisplatin | Cisplatin | 0.340 | 0.741 | 0.241 | 0.286 | 0.378 |
| 5-FU | 5-FU | 0.192 | 0.331 | 0.192 | 0.308 | 0.478 |
| Etoposide | ICB | 0.522 | 0.522 | 0.300 | 0.391 | 0.416 |
| Bleomycin | Bleomycin | 0.374 | 0.522 | 0.411 | 0.760 | 0.515 |
| Gemcitabine | ICB | 0.409 | 0.331 | 0.283 | 0.930 | 0.542 |
| Doxorubicin | ICB | 0.409 | 0.331 | 0.439 | 1.07 | 0.698 |
| Irinotecan | Irinotecan | 0.481 | 0.501 | 0.478 | 1.14 | 0.256 |
| Vinorelbine | Vinorelbine | 0.813 | 0.501 | 1.78 | 1.96 | 1.23 |
| Pemetrexed | Pemetrexed | 1.24 | 0.501 | 2.46 | 2.26 | 1.50 |

**HR Combo vs best mono is the chemo-immunotherapy combination treatment compared to best performing monotherapy calculated from survival analysis

**Figure S1**. **Gating strategies for flow cytometry analysis of myeloid and lymphoid panels.** A) Parent gating for both lymphoid and myeloid panels. B) Gating for myeloid subsets from CD45^+^ cells. Neutrophils: CD11b^+^Ly6C^+^Ly6G^+^; Inflammatory monocytes: CD11b^+^Ly6G^-^Ly6C^hi^; Resident monocytes: CD11b^+^Ly6G^-^Ly6C^lo^; Macrophages: CD11b^+^Ly6C^-^ MHCII^+^CD11c^-^; Conventional dendritic cells: CD11b^+^Ly6C^-^ MHCII^+^CD11c^+^; Plasmacytoid dendritic cells: CD11b^-^Ly6C^+^ MHCII^+^CD11c^+^. C) Gating for lymphoid subsets from CD45^+^ cells. ICOS and Ki67 expression were analyzed on CD335^+^; CD8^+^, CD4^+^Foxp3^-^ & CD4^+^Foxp3^+^ T cells.

**Figure S2. Survival plots of AB1 bearing mice treated with chemo-immunotherapy**. ICB: immune checkpoint blockade therapy (anti-CTLA-4, anti-PD-L1); Cyclo: cyclophosphamide; Gem: gemcitabine. Mantel-Cox survival test; *P < 0.05, **P<0.01,

**Figure S3.** **Survival plots of AE17 bearing mice treated with chemo-immunotherapy**. ICB: immune checkpoint blockade therapy (anti-CTLA-4, anti-PD-L1); Cyclo: cyclophosphamide; Gem: gemcitabine. Mantel-Cox survival test; *P < 0.05, **P ≤ 0.01

**Figure S4. Flow cytometry and bulk RNAseq analysis on additive chemo‑immunotherapies.** A) Representative tumor growth curves (left) and tumor sizes at harvest (right) of AB1/AB1-HA tumor bearing mice for flow cytometry and bulk RNAseq analysis. Data shown as mean ± SD, n = 5 per group. Harvest occurred 2 days after the last dose of anti-PD-L1 therapy. B-D) Frequencies of CD8^+^, CD4^+^Foxp3^-^ and CD4^+^Foxp3^+^ T cells in DLNs (B) and spleens (C), and their activation status (ICOS^+^Ki67^+^) in spleens (D) of chemo/immunotherapy treated mice. Data shown as mean ± SD, flow cytometry data is summary of two independent experiments (n=6 per group). Mann-Whitney U tests corrected for multiple comparisons; *P < 0.05, **P ≤ 0.01.

**Figure S5. 5-FU chemotherapy depletes neutrophils and inflammatory monocytes in DLNs, spleens and tumors.** A) Representative FAC plots of CD45^+^CD11b^+^Ly6C^+^ cells expressing Ly6C and Ly6G in chemo-immunotherapy treated spleens. Gates on the FACS plots represent neutrophils (Ly6C^+^Ly6G^+^), inflammatory monocytes (Ly6C^hi^Ly6G^-^) and resident monocytes (Ly6C^lo^Ly6G^-^). B) Dot plots representing frequencies of neutrophils, inflammatory and resident monocytes of spleens from chemo-immunotherapy groups. Data shown as mean ± SD, summary of two independent experiments (n = 6 per group). Mann-Whitney U tests corrected for multiple comparisons; *P <0.05, **P ≤ 0.01, ***P ≤ 0.001.

**Figure S6. Combination chemo-immunotherapy increases tumor infiltration of CD8^+^ and CD4^+^ helper T cells and decreases CD4^+^ regulatory T cells.** A-B) Frequencies of CD8^+^, CD4 Helper (CD4^+^Foxp3^-^) and regulatory T cells (T_regs_; CD4^+^Foxp3^+^) in chemo-immunotherapy treated tumors analyzed by flow cytometry (A) and CIBERSORTx (B)**.** C) Frequencies of neutrophils, inflammatory monocytes and resident monocytes in chemo-immunotherapy treated tumors analyzed by flow cytometry. D) Proportion of monocytes analysed by CIBERSORTx in chemo-immunotherapy treated tumors. No neutrophils were found in CIBERSORTx analysis. Data shown as mean ± SD, flow cytometry data is summary of two independent experiments (n = 6 per group), RNAseq data is one experiment (n = 5 per group, except PBS and cisplatin n = 4 per group). Mann-Whitney U tests corrected for multiple comparisons; *P < 0.05, **P ≤ 0.01

**Figure S7**. **Granzyme B and IFN𝛾 expression on CD8 T cells after chemo-immunotherapy.** A-B) Frequency (left) and number (right) of CD8^+^GzmB^+^ T cells (A) and CD8^+^IFN𝛾^+^ T cells (B) in draining lymph nodes (DLN; top) and tumors (TUM; bottom) after chemotherapy and/or ICB. C) Representative FACs plot of CD8^+^ T cells from an ICB treated tumor expressing GzmB (top) and IFN𝛾 (bottom). GzmB: granzyme B; DLN: draining lymph node; TUM: tumor. **P<0.01.

**Figure S8.** **Additive chemo-immunotherapies upregulate immune related pathways in the tumor microenvironment.** A) Unsupervised hierarchical clustering of the top 200 differentially expressed genes from cisplatin+ICB treatment groups. B) Downregulated Reactome pathways in 5-FU+ICB compared to ICB treated tumors. C) Top 10 upregulated Reactome pathways in 5-FU+ICB when compared 5-FU. D-E) Top 10 upregulated KEGG (D) and Reactome (E) pathways in cisplatin+ICB compared to cisplatin alone. Multiple comparisons corrected using Bonferonni-Hochberg method. Significance denoted by P<0.05. D) GSEA displaying top hallmark gene sets significantly (q < 0.25) enriched in cisplatin+ICB compared to cisplatin, 5-FU+ICB compared to 5-FU and 5-FU+ICB compared to ICB. For each GSEA plot, a positive normalized enrichment score (NES) indicates that specific gene set is enriched in the combination chemo-immunotherapy treated tumor compared to the monotherapy. CIS+ICB, cisplatin+ICB; CIS, cisplatin; B-H, Bonferonni-Hochberg.
